# Cytotoxicity of Etch-and-Rinse, Self-Etch, and Universal Dental Adhesive Systems in Fibroblast Cell Line 3T3

**DOI:** 10.1155/2017/9650420

**Published:** 2017-01-10

**Authors:** Yasmine Mendes Pupo, Cintia Fernanda de Freitas Bernardo, Francielly Fernanda de Freitas A. de Souza, Milton Domingos Michél, Camila Nunes de Morais Ribeiro, Sandro Germano, Daniela Florencio Maluf

**Affiliations:** ^1^Department of Restorative Dentistry, Federal University of Parana (UFPR), Curitiba, PR, Brazil; ^2^School of Dentistry, Tuiuti University of Parana (UTP), Curitiba, PR, Brazil; ^3^Department of Materials Engineering, State University of Ponta Grossa (UEPG), Ponta Grossa, PR, Brazil; ^4^Department of Biomedicine, Tuiuti University of Parana (UTP), Curitiba, PR, Brazil; ^5^Department of Pharmacy, Federal University of Parana (UFPR), Curitiba, PR, Brazil

## Abstract

The aim of this study was to evaluate in fibroblast cultures the direct cytotoxic effects of etch-and-rinse, self-etch, and universal adhesive systems. The sterile glass cover slips (*n* = 3) were then immersed in culture medium to obtain the eluates for the experimental groups: (1) Adper™ Single Bond 2; (2) Ambar; (3) Adper™ Scotchbond™ Multi-Purpose; (4) Scotchbond™ Universal; (5) Ambar Universal; and (6) OptiBond All-In-One. As a negative control, sterile glass cover slips were immersed in culture medium only. After 24 h, the eluate obtained was applied on fibroblast culture. Cell viability and cell morphology were evaluated by MTT assay and SEM, respectively. Data were analyzed by Kruskal–Wallis and Mann–Whitney tests (*α* = 0.05). All adhesive systems except universal reduced cell viability in 3T3 cells to between 26.04% and 56.57%, and Scotchbond Universal and Ambar Universal reduced cell viability to 2.13% and 3.57%, respectively, when compared to the negative control. Cytoplasmic membrane shrinkage and cell-free areas with residual membrane fragments from dead cells were observed. In conclusion, improvements in universal adhesive system formulations and their mechanisms of action are not accompanied by increased toxicity compared with those in other systems, warranting commitment to the use of these dentin-pulp complexes.

## 1. Introduction

The evolution of technologies for clinical procedures in dentistry has led to a wide variety of marketed materials. However, in addition to the esthetic care properties and durability of these products, the evaluations of biocompatibility with dental structures are required to optimize compatibility of pulp-dentin tissue complexes [1]. Light composite materials are commonly used in restorative dentistry, and the field of adhesive dentistry encompasses the use of these materials in conjunction with adhesive systems for the restoration or reanatomization of lost dental tissues and for shape changes.

To improve the retention of restorative procedures and to seal tooth-restoration interfaces from microorganisms, adhesive systems are recommended because they improve the contact between resin-based restorative materials and the walls of prepared cavities [2, 3]. However, previous studies have reported that there are various substances with biological effects that are potentially toxic following release from adhesive systems [4]. Numerous materials have been developed for dental applications. However, more tissue-friendly materials remain desired, and the biocompatibility of dental materials is an increasingly important area of research. Although the severity of adverse effects varies, the risks of toxicity remain an important consideration for materials that are placed in contact with oral tissues [3].

Adhesive systems commonly comprise bifunctional monomers and hydrophobic and hydrophilic monomers, which contain carboxylic acid- or phosphoric acid-derived radicals and/or added organic or mineral acid derivatives. Moreover, these monomeric components are present in solvents, such as water, alcohol, and acetone, and also in aromatic amines and filler particles [5]. Time-dependent cytotoxicity of the monomers hydroxymethyl methacrylate (HEMA), bisphenol A diglycidyl dimethacrylate (Bis-GMA), and urethane dimethacrylate (UDMA) has been shown in deep cavities and direct contact with pulp tissue [6]. Consequently, basic cellular functions, such as proliferation, enzyme activity, and mitochondrial respiration, are reportedly compromised, with concomitant changes in cell morphology and membrane integrity [6]. To provide broader indications, other components have been added to universal adhesive systems, and these may cause changes in the biological behaviors of dentin-pulp complexes.

With the evolution of adhesive systems with various components, prior removal of the* smear layer* using conventional approaches has been made redundant by self-etching approaches that maintain the smear layer as part of the adhesion substrate [2]. Previous studies recommend selective etching of enamel margins to accommodate self-etching adhesives that do not allow conditioning of the enamel at the same depth as with phosphoric acid before application in two steps [7]. Accordingly, this approach has been established by new universal adhesives, which have similar composition to that of one-step self-etching primer adhesives. In these primer adhesives, the methacrylic monomers found in total-etch adhesives have been replaced by functional monomers, such as methacryloyloxydecyl dihydrogen phosphate (MDP), leading to characteristic acidic functions and subsequent chemical adhesion.

Due to standardization and reproducibility of cytotoxicity determinations in cell cultures,* in vitro* cytotoxicity assays are suitable for assessments of biocompatibility of adhesive systems containing MDP and can be considered prerequisite for understanding the biological risks of these materials during initial setting. Accordingly,* in vitro* tests using cell cultures can be used to rapidly generate sensitive, inexpensive, convenient, and repeatable material classifications [4, 8]. Therefore, the aim of this study was to evaluate the cytotoxic effects of etch-and-rinse, self-etch, and universal adhesive systems. The hypothesis was that universal dentin adhesive containing MDP may be less cytotoxic on fibroblasts than conventional and self-etch dentin adhesives systems.

## 2. Material and Methods

Included adhesive systems are described in Table 1 and included (1) two-step etch-and-rinse, Adper Single Bond 2 (SB, 3M ESPE; St. Paul, MN, USA); (2) two-step etch-and-rinse, Ambar (AM, FGM; Joinville, SC, Brazil); (3) three-step etch-and-rinse, Adper Scotchbond Multi-Purpose (MP, 3M ESPE; St. Paul, MN, USA); (4) two-step etch-and-rinse or one-step self-etch, Scotchbond Universal (SBU, 3M ESPE; St. Paul, MN, USA); (5) two-step etch-and-rinse or one-step self-etch, Ambar Universal (AMU, FGM; Joinville, SC, Brazil); and (6) One-step self-etch, OptiBond All-In-One (OPT, Kerr; Orange, CA, USA).

### 2.1. Material Preparation

Aliquots (10 *μ*L) of the adhesive systems (SB, AM, SBU, AMU, and OPT) were pipetted in sextuplicate into sterile circular microscopy coverslips (G-13C100) of 13 mm diameter and 0.13 mm thickness (Glasscyto, Bioslide Technology, Walnut, CA, USA). For Scotchbond Multi-Purpose (MP), 5 *μ*L primer and 5 *μ*L bond were pipetted. The aliquots were light-cured with LED (Valo, Ultradent Products Inc.; South Jordan, USA; irradiance: 1400 mW cm^2^) for 10 s. Coverslips containing adhesive systems were disposed into sterile 6-well plates where 3 mL per well of culture medium RPMI supplemented with 10% fetal bovine serum (FBS) and antibiotics (penicillin/streptomycin 100 IU/100 *μ*g·mL^−1^) was added. The plates were incubated at 37°C for 24 hours. Subsequently, culture media containing leached components of the adhesive systems were collected and sterilized by filtration through 0.22 *μ*m membrane filters to obtain sterile eluates for cell application. The same procedure was conducted with coverslips in the absence of adhesive system to characterize the negative control.

### 2.2. Cell Viability Analysis

Cell viability was determined according to mitochondrial activity in proliferation assays. In these assays, soluble 3-(4,5-dimethylthiazol-2-yl)-2,5-diphenyltetrazolium bromide (MTT) salt is converted to insoluble formazan crystals by mitochondrial succinate dehydrogenase (SDH) in viable cells, dissolved in dimethyl sulfoximine (DMSO), and formazan concentrations are measured spectrophotometrically at 570 nm.

The cells were concentrated in 1 mL of RPMI containing 10% SFB and antibiotics, and the numbers of viable cells were determined using the trypan blue method. Considering cell viability of >90%, suspensions of 3 × 10^5^ cells were seeded into 96-well plates (100 *μ*L/well). Plates were incubated for 24 h at 37°C in 5% CO_2_ to allow cell adhesion. Culture medium was then removed and cells were treated with the conditioned media from test materials in triplicate at 37°C overnight. Control cells were treated with nonconditioned medium. Subsequently, the cells were washed with 200 *μ*L of sterile PBS (37°C) in duplicate, and 100 *μ*L of MTT reagent solution (Sigma-Aldrich) in sterile PBS (0.5 mg/mL) was added to each well and incubated at 37°C for 4 h. Culture medium containing MTT was then replaced with 100 *μ*L of pure DMSO to dissolve formazan crystals. Cell viability was then evaluated spectrophotometrically at 570 nm using a microplate reader (ELX 800, BioTek Instruments; Winooski, VE, USA). The MTT assay was conducted three times to confirm reproducibility. Absorbance data were expressed relative to the control group and percent viability was calculated. Data are presented as mean ± standard deviations.

### 2.3. Cell Morphology Analysis Using Scanning Electron Microscopy (SEM)

3T3 fibroblast cells were seeded at 6 × 10^4^ cells/well in 6-well microplates containing coverslips (13 mm diameter and 0.13 mm thickness) for sterile microscopy. Cells were treated with conditioned media from adhesive systems, which were light-cured for 10 s and stored for 24 h, as described above. After treatment, cells that remained attached to glass coverslips were fixed with 1 mL of 2.5 glutaraldehyde solution for 24 h and were then dehydrated using an ethanol series of 30, 50, 70, 95, and 100% in concentration. Cells were then maintained in colloidal silica for 24 h and were sputter-coated with gold/palladium in a metallizer (Shimadzu, Kyoto, Japan). Cell morphology of 3T3 fibroblasts was then assessed using SEM (Shimadzu).

### 2.4. Data Analysis

Data from MTT assays were expressed as percent viability relative to the negative control (culture medium; 100%). Statistical analyses were performed using SPSS version 21 and differences were identified using Kruskal–Wallis and Mann–Whitney nonparametric tests and were considered significant when *p* < 0.05.

## 3. Results

### 3.1. Cytotoxic Effects of Adhesives

**Figure 1:**
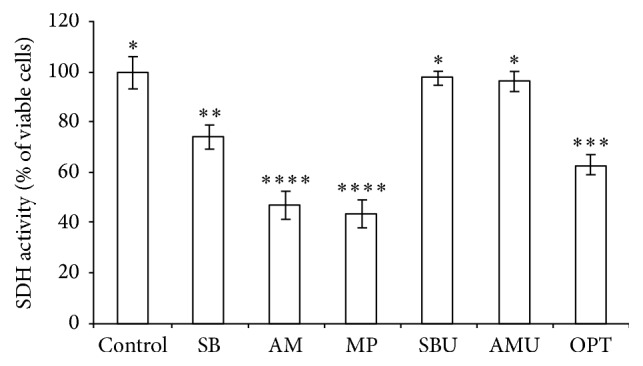
Percentage (%) of cell viability of 3T3 cells; columns with the same quantity asterisks did not differ significantly (Mann–Whitney, *p* > 0.05).

The treatment with the adhesives resulted in cell death average of 2.13% to 56.57%, demonstrating a moderate cytotoxic effect of the tested systems (Figure 1).

### 3.2. SEM Analyses of Cell Morphology

**Figure 2:**
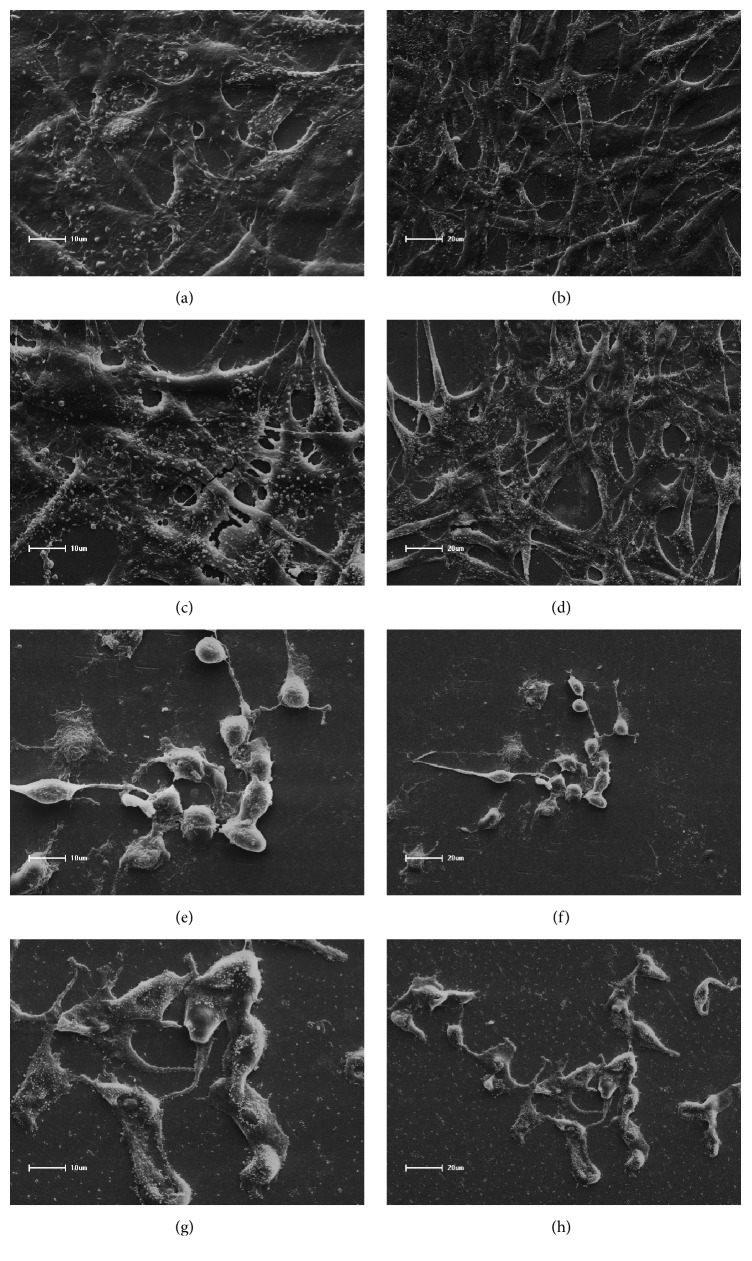
Cell morphology in scanning electron microscopy (SEM) of 3T3 fibroblast cells. (a) Positive control group, large numbers of 3T3 fibroblasts with fusiform morphology, ×1000; (b) positive control group, spindle-shaped appearance with some cytoplasmic processes originating from membranes covering the glass substrate surface, ×500; (c) SB, large numbers of fibroblasts with normal morphology, ×1000; (d) SB, fusiform cells, uninucleation, and long and thin cytoplasmic membrane processes, ×500; (e) AM, altered morphology, rounded shapes, and small sizes, ×1000; (f) AM, cytoplasmic membranes and debris from dead cells, ×500; (g) MP, severe cytotoxic effects, ×1000; (h) MP, reduced numbers of cells, membrane rupture, and apoptosis, ×500.

**Figure 3:**
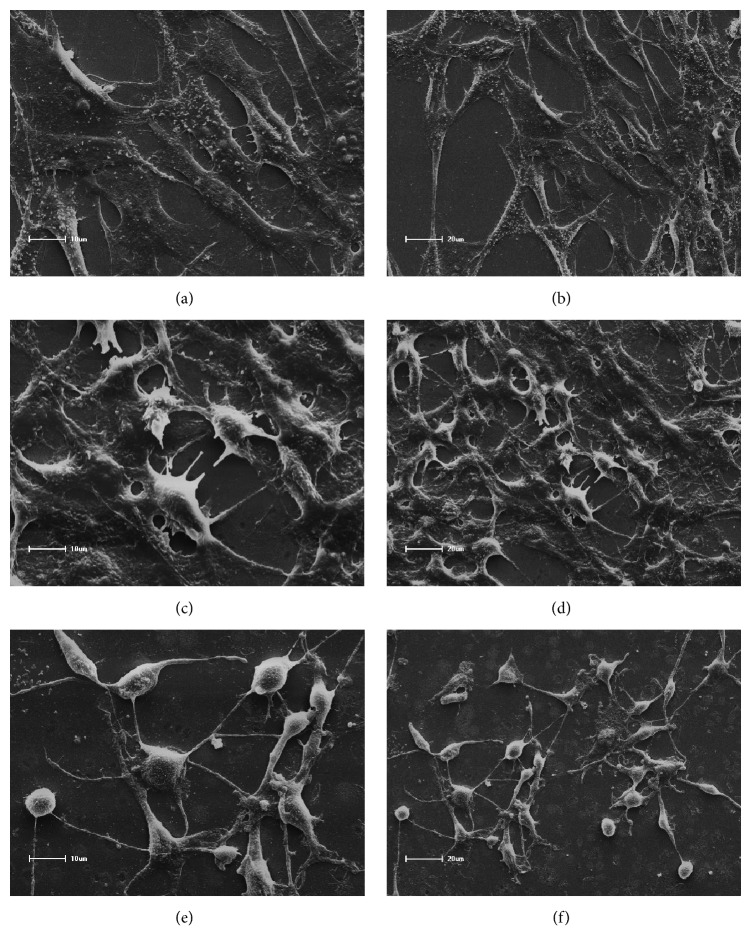
Micrographs from SEM analyses of 3T3 fibroblasts. (a) SBU, no changes in morphology compared with those in the control group, ×1000; (b) SBU, abundant cytoplasm with numerous elongated thin cytoplasmic projections adhering to the glass surface, ×500; (c) AMU, similar morphology to the control group, ×1000; (d) AMU, long, thin cytoplasmic membrane derived prolongations, ×500; (e) OPT, altered morphology with ill-defined cellular limits, suggesting cell necrosis, ×1000; (f) OPT, morphological changes and ruptured membranes in 3T3 cells, ×500.

SEM micrographs of 3T3 fibroblasts after treatment with adhesive eluates are presented in Figures 2(a)–2(h) and 3(a)–3(f). These analyses demonstrated fusiform morphology of adherent positive control 3T3 cells (exposed only to culture medium), with large numbers of elongated cells on the coverslip surface showing spindle shapes and cytoplasmic membrane processes (Figures 2(a) and 2(b)).

Following exposure to Adper Single Bond 2 (Figures 2(c) and 2(d)), large numbers of cells with regular morphology were observed, and near confluent cells showed abundant cytoplasm with numerous elongated, fine cytoplasmic projections adhering to glass surfaces. Similar characteristics were observed following exposure to Scotchbond Universal and Ambar Universal (Figures 3(a), 3(b), 3(c), and 3(d)). However, severe cytotoxic effects of the conditioned media from some experimental materials were observed in fibroblast 3T3 cells (Figures 2(e), 2(f), 2(g), 2(h), 3(e), and 3(f)). Accordingly, fewer cells were attached to the substrate. The analysis of cell morphology by SEM revealed the occurrence of death with consequent detachment from the glass substrate of some cells. Furthermore, apoptosis was characterized by condensation of the nucleus and cytoplasm, followed by membrane-bound fragmentation of the cell [5, 9, 10].

## 4. Discussion

Efficacy and biocompatibility assessments are critical for the clinical validation of dental materials. Thus, several* in vitro* studies have investigated the cytotoxicity of dental adhesive systems and their components [4], and according to Williams [11] these are more reproducible and convenient than* in vivo* tests. Dental adhesives generally comprise complex mixtures of crosslinked and functional hydrophilic and hydrophobic monomers in solvents, such as acetone, ethanol, and/or water. Although these adhesives contain low concentrations of initiators and inhibitors of polymerization reactions, previous studies have shown that monomers can interfere with adaptive responses and can aggressively deplete vital cellular functions by generating oxidative stress and exhausting antioxidant defense mechanisms [12].

Conventional or total-etch adhesive systems require demineralization of dental enamel and dentin substrates using phosphoric acid conditioning before application [13]. The formation of hybrid layers during total conditioning with adhesive systems relies on superficial dentin demineralization by inorganic acids, which exposes collagen fibrils to infiltration by hydrophilic monomers [14]. However, dentin humidity continues to hamper the use of conventional adhesive systems [15] by preventing complete infiltration throughout the collagen matrix mesh, resulting in outbreaks for degradation of the bonding interface and higher rates of postoperative sensitivity [13]. To address the detriments of dentin humidity, self-etching adhesive systems in which monomer acids demineralize and infiltrate substrates simultaneously have been developed, precluding the use of acid in a separate step to produce porosity of the substrate [16]. Previous studies have shown lower cytotoxicity of self-etch adhesive systems and better responses in histological tissues than that following use of total-etch adhesive systems [4]. The provision of a bond that is effective for various dental substrates is the main challenge of current dental adhesives [14]. In particular, one-step self-etch adhesive has been introduced and classified as “universal” or “multimode” [17]. This multiapproach capability enables the clinician to apply the adhesive with the so-called selective enamel etching technique that combines the advantages of the etch-and-rinse technique on enamel, with the simplified self-etch approach on dentine with additional chemical bonding on remnant carbonated apatite crystallites in those bonding substrates [18].

The present study shows that adhesive systems can have metabolic effects in 3T3 fibroblasts, which were chosen to assess the toxicity of monomer materials due to the ease and speed of cell growth, and they are in line with ISO recommendations for evaluations of biological responses to dental materials [19]. The present data show that whereas the Ambar (47.03%) and Adper Scotchbond Multi-Purpose (43.43%) had severe cytotoxic effects, Adper Single Bond 2 (73.96%) and OptiBond All-In-One (62.94%) were less cytotoxic. However, the Scotchbond Universal (97.44%) and Ambar Universal (96.05%) adhesive systems were better tolerated by 3T3 fibroblasts, offering greater security to dentin-pulp complex. Adhesive toxicity varied among the present adhesive systems and was likely related to the presence of residual monomers in eluates.

Conventional self-etching adhesives have greater quantities of hydrophobic monomers than universal adhesives, likely leading to greater toxicity. Accordingly, the present MTT experiments reflected the presence of residual monomers in eluates from total-etch adhesives. Residual monomers in the present adhesives included HEMA, Bis-GMA, UDMA, and MDP. These compounds are known to change cellular microenvironments by inducing the formation of reactive oxygen species (ROS) and depleting antioxidants, such as glutathione. Moreover, increased ROS levels are directly related to the control of cell death by antioxidant genes and proteins [20]. HEMA is a monomer that improves dentin bonding strength. Despite being less toxic, HEMA has a low molecular weight and carries a hydroxyl group with hydrogen binding affinity and is easily released in aqueous solution [5, 6, 21, 22]. This monomer suppresses the growth of many cells types, induces delays in cell cycle progression of primary fibroblasts by increasing ROS levels, and can also activate apoptosis [3]. Similarly, Bis-GMA alters cell cycle progression, elevates oxidative stress, and induces apoptosis in a concentration-dependent manner. However, hydrophobic monomers of Bis-GMA have a high molecular weight and chemical features, such as high viscosity, low volatility, and low polymerization shrinkage, which result in stricter resins with lower susceptibility to hydrolytic media [5, 22]. UDMA is also a viscous hydrophobic monomer, and its high molecular weight leads to relative resistance of composite resins. However, UDMA induces cellular changes at low concentrations, leading to pathological phenotypes and cell death [23].

Adhesive systems also include other components that alter their properties, and the present universal adhesive system contains functional monomers, such as MDP, Vitrebond™ Copolymer, and silane. MDP is a long carbon chain monomer that contributes hydrophobicity and hydrolytic stability [12]. Moreover, the presence of silane in this adhesive allows direct application to the crowns of grafts [5]. Thus, cell viability in the presence of universal adhesive systems likely reflects the presence of multiple components. A previous study also demonstrated influences of monomer types and interactions on cytotoxic effects [1], reflecting differences in cytotoxicity concentrations, types and interactions of monomers, types of solvents, and molecular weights. However, further studies are required to specifically define relationships among adhesive components and interactions and cytotoxicity.

In the present SEM analyses, large numbers of cells with normal morphology and cytoplasmic projections on coverslip surfaces were observed in the presence of SB, SBU, and AMU adhesives. In contrast, the presence of adhesives AM, MP, and OPT led to reduced numbers of attached cells, rounded and small morphology, and rupture of cytoplasmic membranes, reflecting widespread cell death [5, 9, 10].

One of the limitations of this study is that it is an* in vitro* experiment; thus it may not directly reflect the clinical situation. Therefore, the use of more clinically relevant cells is important [24]. The universal dental adhesives have few studies reporting their clinical and biological performances [8, 25]. In this study, universal dental adhesive showed the highest fibroblast viabilities according to the MTT assay. Hence, this universal adhesive offers a good alternative for specific cases and has technical simplicity. However, further longitudinal studies are required to investigate the effects of other components following application using various techniques. These observations encourage studies in isolated dental pulp stem cells.

## 5. Conclusions

Under the limitations of this study, improvements of formulations and the mechanisms of action of universal adhesive systems did not produce greater toxicity when compared with other systems.

## Figures and Tables

**Table 1:** Test materials, classifications, and compositions.

Adhesive system (manufacturer)	Lot number	Classification	Composition
Adper Single Bond 2 (3M ESPE)	#N508311	Two-step etch-and-rinse	Bis-GMA, HEMA, polyacrylic acid, poly(itaconic acid), water, ethanol, dl-CQ, silica (10% wt).
Ambar (FGM)	#240215	Two-step etch-and-rinse	UDMA, HEMA, 10-MDP, hydrophilic methacrylated monomers, ethanol, silica nanofiller, photoinitiators, coinitiators, stabilizers.
Adper Scotchbond	Primer: #N560292	Three-step etch-and-rinse	Primer: HEMA, polyalkenoic acid polymer, water.
Multi-Purpose (3M ESPE)	Bond: #N551363		Bond: Bis-GMA, HEMA, tertiary amines, photoinitiator.
Scotchbond Universal (3M ESPE)	#569736	Two-step etch-and-rinse or one-step self-etch	Bis-GMA, HEMA, ethanol, water, silane treated silica, 2-propenoic acid 2-methyl-, reaction products with 1, 10-decanediol and phosphorous oxide (P_2_O_5_), copolymer of acrylic and itaconic acid, CQ, 4-dimethylaminobenzoate, toluene.
Ambar Universal (FGM)	#030815	Two-step etch-and-rinse or one-step self-etch	UDMA, HEMA, 10-MDP potentiated, hydrophilic methacrylated monomers, ethanol, silica nanofiller, photoinitiators, coinitiators, stabilizers.
OptiBond All-In-One (Kerr)	#5125872	One-step self-etch	GPDM, HEMA, GDMA, Bis-GMA, water, 2.5–3 acetone, ethanol, CQ, silica filler, sodium hexafluorosilicate.

Bis-GMA, bisphenol A diglycidyl methacrylate; HEMA, 2-hydroxyethyl methacrylate; CQ, camphorquinone; UDMA, urethane dimethacrylate; 10-MDP, 10-methacryloyloxydecyl dihydrogen phosphate; GPDM, glycerol phosphate dimethacrylate; GDMA, glycerol dimethacrylate.
